# Apolipoprotein E Gene Polymorphism in Iranian Coronary Atherosclerosis Patients Candidate for Coronary Artery Bypass Graft

**Published:** 2013-07

**Authors:** Mohammad Mehdi Heidari, Seyed Khalil Foruzannia, Mehri Khatami, Mehdi Hadadzadeh, Mahmoud Emami Meybodi

**Affiliations:** 1 Department of Biology, Science School, Yazd University, Yazd, Iran; 2 Department of Cardiac Surgery, Afshar Hospital, Shahid Sadoughi University of Medical Sciences, Yazd, Iran

**Keywords:** Apolipoprotein E, Coronary Atherosclerosis, Polymorphism, Restriction Isotyping

## Abstract

***Objective(s)*** ***:*** Apolipoprotein E genotype (*APOE*) polymorphism affects lipid levels and coronary artery disease (CAD) risk. The aim of this study was to study the association of the Apolipoprotein E **genotypes** with coronary artery disease in the Iranian population.

***Materials and*** ***Methods:*** The Apolipoprotein E genotype in DNA samples extracted from 66 CAD+ patients and 61 control subjects by restricting enzyme digestion of amplified exon 4 *APOE* gene was determined.

***Results*** *:* The ε3 allele was found at similar frequency in control subjects (88.5%) and atherosclerosis patients (83.3%) (*P*=0.314). Our results showed that the frequency of the ɛ3/ɛ3 and ε3/ε4 genotypes increased in three-vessel-disease patients and the frequency of ɛ2/ɛ2 genotype increased in one-vessel-disease patients.

***Conclusion*** *:* ɛ3/ɛ3 and ɛ3/ɛ4 genotypes are suggested to be predisposing factors, which, in combination with environmental factors, may trigger the degree of luminal narrowing. The possible mechanisms remain elusive and require further studies.

## Introduction

Atherosclerosis is a lifelong process that begins early in life and results in clinically manifest coronary artery disease in middle age and later. Risk factors for coronary heart disease in adults (age, smoking, and high serum lipoprotein cholesterol levels) are associated with the extent and severity of atherosclerosis (1, 2). Common genetic variants of human apolipoprotein (apo) E are also associated with differences in lipid risk factors and atherosclerosis.

Apolipoprotein E (apoE) is a 299-amino acid plasma protein involved in cholesterol transport and is found in chylomicrons, very low density lipoprotein, intermediate-density lipoprotein, and high-density lipoprotein (3, 4). ApoE plays an important role in the metabolism of these lipoproteins by binding to the low-density lipoprotein (LDL) receptor in hepatic and extrahepatic tissues and a putative apoE receptor or LDL receptor-related protein.

The Apolipoprotein is encoded by a 4 exon gene located on the long arm of chromosome 19 (5). The *APOE* gene spans approx 3.7 kb and has been cloned and sequenced (5, 6). Like other apolipoprotein genes, it consists of four exons separated by three introns, with most of the protein-coding sequence contained in exon 4. The length of the mRNA is approx 1100 nucleotides. The expression of the *APOE* gene is regulated by multiple positive and negative elements within its promoter region (5, 7).

The polymorphic nature of *APOE* was first described about 20 years ago, and three common isoforms-E2, E3, and E4-are recognized. These are encoded by three common alleles- ε2, ε3, and ε4-that are expressed codominantly, generating six possible phenotypes-E2/2, E2/3, E2/4, E3/3, E3/4, and E4/4 (4, 8, 9). E3 is the most common form in all populations studied. In typical Caucasian populations, ε3 is the most common allele, occurring in more than 75% of chromosomes. The average frequencies of ε2 and ε4 are 8 and 15%, respectively (4).

The ε4 allele is a dose-dependent risk factor for Alzheimer’s disease. It is also associated with higher total serum cholesterol and LDL cholesterol levels and with increased risks of atherosclerosis and ischemic heart disease (9). The aim of this work was to study the association of the Apolipoprotein E genotypes with coronary artery disease in the Iranian population and to evaluate the role of apolipoprotein E gene polymorphism as a predisposing factor for atherosclerosis patients.

**Table 1:** The Summary of the clinical and genetic analysis of coronary atherosclerosis patients

	Patients (n= 66)	Controls (n=61)
Male gender (%)	35	32
Mean age± SD (years)	52.5±7.9	51.2± 7.1
Smokers	22.7%	7.5%
Body mass index (kg/m^2^)	25.4±1.9	25.1±1.7
Cholesterol, mg/dl	206.8±54.5	181.6±39.3
LDL-C, mg/dl	123.6±45.6	114.6±45
HDL-C, mg/dl	40.5±8.5	49.4±12.7
TGs, mg/dl	198.8±106.5	148.5±92.1

## Materials and Methods

***Subjects***

66 patients and 61 healthy controls were investigated for age, sex and ethnicity. The clinical characteristics and data for current medication usage in the two groups are summarized in Table 1. Selective coronary angiography was performed by a qualified cardiologist using the standard Judkins technique in several planes. The films were analyzed independently by a cardiologist and a radiologist.

Subjects were divided into two groups: CAD group with normal coronary artery and CAD^ +^ group with significant lesions (>50% narrowing of luminal diameter) in one, two, or three vessels (LAD, LCX, and RCA) that were candidate for CABG (*Coronary Artery Bypass Graft*).

All of the patients and control group were informed about the aims of the study and gave their informed consents to the genetic analysis. Informed consent, blood samples, and clinical evaluations were obtained from all of the participating family members.

*DNA analysis for the detection of ApoE genotypes*

**Figure 1:**
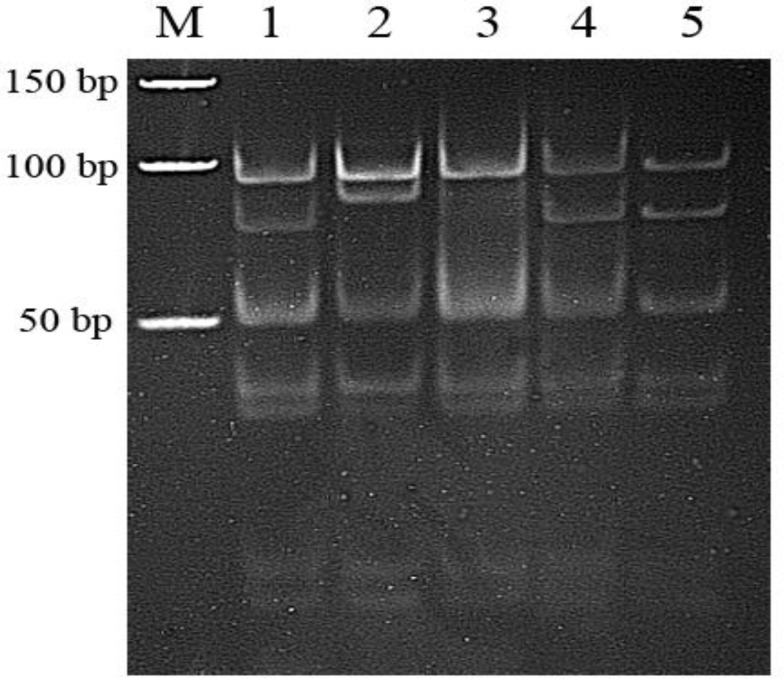
Electrophoretogram showing PCR-RFLP in the apolipoprotein E. Lane 1, 4, 5: ɛ3/ɛ 4 and 3: ɛ3/ɛ3; lane 2: ɛ2/ɛ2 genotype

DNA was isolated from peripheral blood samples using a DNA extraction kit (DNAfast Kit-Genfanavar-ran, Tehran, Iran). PCR used oligonucleotide primers (Takapouzist, Iran) that flank positions 112 and 158 in exon 4 of the *APOE* gene (forward primer 5'-TAAGCTTGGCACGGCTGTCCAAGGA-3'; reverse prim-

er 5'- ACAGAATTCGCCCCGGCCTGGTACAC-3'). Each amplification reaction contained 100 ng total DNA, 10 pmol of each primer, 2.5 Mm MgCl2, 200 µM, 10% dimethyl sulfoxide, and 1 U Taq DNA polymerase (Roche Diagnostics, Mannheim, Germany) in a final volume of 25 µl. Each reaction mixture was heated at 95°C for 5 min for denaturation and subjected to 30 cycles of amplification by primer annealing (60°C for 1 min), extension (70°C for 2 min), and denaturation (95°C for 1 min) (10). After PCR amplification, in a final volume of 20 µl, 10 µl PCR product digests with 1 µl HinPI (5 units/µl) (Fermentas, Russia) and 2 µl HinPI buffer (3 hr at 37°C). Each reaction mixture was loaded onto 8% polyacrylamide nondenaturing gels and electrophoresed for 5 hr under constant current (20 mA). After electrophoresis, gels were treated with ethidium bromide (0.2 mg/l) for 10 min, and visualized using an Uvitec transilluminator (Syngene, England) Figure 1.

***Data analysis***

Levels of the quantitative variables are presented as mean±SEM. Frequency data between normal controls and patients were compared using Pearson's chi-square test. The GraphPad Prism software was used for statistical analaysis, with *P- *values below 0.05 considered indicative of statistical significance.

## Results

The healthy controls were selected to have closely similar ranges of age and BMI with the CAD+ patients. All patients were under 65 years of age (52.5±7.9 years). The corresponding figures for the healthy controls were 51.2± 7.1 years. 

Coronary angiography revealed 66 patients (CAD+ group) with one-vessel (n=l3), two-vessel (n=25), or three-vessel (n=28) involvement of coronary arteries and 61 patients (CAD- group) with no angiographically identified narrowing.

**Table 2:** Apolipoprotein Eof CAD^+^ patients and controls

Allele	Patients					
	One- V	Two- V	Three- V	Total	Controls	*P*-value
ɛ 2	8	4	4	16(12.12%)	14 (11.47%)	0.873
ɛ 3	18	44	48	110 (83.3%)	108 (88.5%)	0.314
ɛ 4	0	2	4	6 (4.5%)	0 (0%)	0.017
ɛ 2/ɛ 2	4	2	2	8 (12.12%)	7 (10.6%)	0.910
ɛ 3/ɛ 3	9	21	22	52 (78.8%)	54 (81.8%)	0.140
ɛ3/ ɛ 4	0	2	4	6 (9.1%)	0 (0%)	0.016
Total	13	25	28	66	61	

V; Vessel

The frequency of the Apolipoprotein E gene in the two study populations are given in Table 2. The predominant allele in control subjects and patients were ε3. The ε3 allele was found at similar frequency in control subjects (88.5%) and atherosclerosis patients (83.3%) (*P*=0.314). The observed homozygosity values for the prevalent allele are close to equilibrium predictions. Other alleles of ε2 and ε4 were detected, but no other allele was found in the control subjects and Atherosclerosis patients.

**Figure 2:**
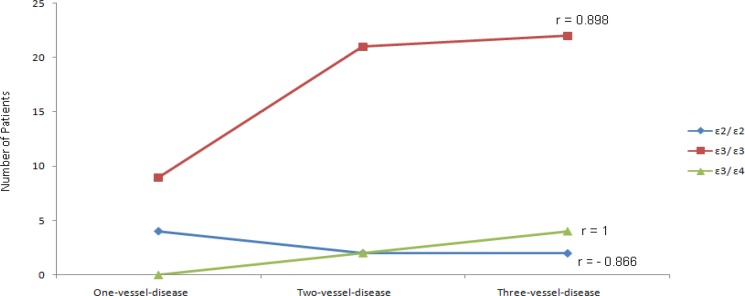
The Correlation between Apolipoprotein E genotype and the degree of luminal narrowing in CAD+ patients

Also, the results of the present study show that the ε3/ε3 and ε3/ε4 genotypes have a statistically significant correlation with the degree of luminal narrowing and a statistically significant inverse correlation between ε2/ε2 genotype with the degree of luminal narrowing (Figure 2).

## Discussion

ApoE plays an important role in the metabolism of triacylglycerol-rich lipoproteins and is described as an important determinant of serum cholesterol level. Carriers of the allele E4 have a higher level of plasma low density lipoproteins (LDL) cholesterol and carriers of the allele E2 have a lower level of LDL cholesterol compared to carriers of the common ε3/ ε3 genotype (11). A significant heterogeneity in *APOE* polymorphism frequencies was observed among different ethnic groups in these studies (12, 13). 

This study reports the *APOE* genotypes in 66 patients with coronary arthrosclerosis patients. The distribution of the ε3 allele in CAD+ group showed no notable difference from that in control subjects (Table 2), but the frequency of ε4 in CAD+ group was higher than CAD- group (*P*= 0.017). Wilson *et al* showed that the ε4 allele may portend the greatest risk for CAD+ (14). The ε2 allele clearly is associated with lower involvement in CAD+ and CAD- groups. 

Recent study in Turkey demonstrated that ε3 allele is the most common allele in individuals of Turkish descent. In this study, ε4 and ε2 allele frequencies were 7.9% and 6.1%, respectively (15). Of the various *APOE* genotypes, the ε3/ε3 was the most frequent genotype. Similar results were reported in other studies (-). 

Previously, studies in the pediatric population have shown that the apoE phenotype strongly influences the lipid profile in childhood (19, 20). Wang *et al* analyzed *APOE* gene polymorphisms in 62 subjects with carotid artery stenosis confirmed by angiography and in 71 healthy subjects, and his results suggested that *APOE* gene polymorphism is correlated with carotid artery stenosis and changes of lipoproteins, and that the gene encoding ε4 is a risk factor for atherosclerosis formation (21).

These results show that the ε3/ε3 and ε3/ε4 genotypes have an association with the degree of luminal narrowing (r =0.898 and r=1, respectively) and a statistically significant inverse correlation between ε2/ε2 genotype with the degree of luminal narrowing (r=-0.866) (Figure 2). 

## Conclusion

These findings suggested that the frequency of the ε3/ε3 and ε3/ε4 genotypes increased in three-vessel-disease patients and the frequency of ε2/ε2 genotype increased in one-vessel-disease patients. We suggest that ε3/ε3 and ε3/ε4 genotypes are predisposing factors which in combination with environmental factors may trigger the degree of luminal narrowing. The possible mechanisms remain elusive and require further studies. 
